# The Shift in Delivery of Care from Hospital to Community Care Settings: What Changes in Terms of Healthcare Workers’ Exposure to Violence

**DOI:** 10.3390/healthcare14070896

**Published:** 2026-03-31

**Authors:** Ettore Minutiello, Pietro Marraffa, Manuela Martella, Alessia Pascarella, Stefano Savigni, Gianfranco Politano, Maria Michela Gianino

**Affiliations:** 1 Hospital Directorate, Maria Vittoria-Amedeo di Savoia Hospital, Turin Local Health Authority, 10143 Turin, Italy; ettore.minutiello@aslcittaditorino.it; 2 Department of Sciences of Public Health and Paediatrics, University of Turin, 10126 Turin, Italy; manuela.martella@unito.it (M.M.); alessia.pascarella@unito.it (A.P.); stefano.savigni@unito.it (S.S.); mariola.gianino@unito.it (M.M.G.); 3 Department of Control and Computer Engineering, Polytechnic of Turin, 10138 Turin, Italy; gianfranco.politano@polito.it

**Keywords:** community settings, hospital, violence, healthcare workers

## Abstract

**Highlights:**

**What are the main findings?**

- Workplace violence against healthcare workers appears to be less frequent in community settings than in hospitals; however, incident reports in community settings increased over time. This trend may represent changes in reporting behaviour, a true increase in incidence, or a combination of both.
- Specific risk factors in community settings include female healthcare workers and the timing of aggression (afternoon for non-physical, morning for physical aggression).

**What are the implications of the main findings?**

- Safety measures and prevention strategies may need adaptation to community-based care contexts, which can differ operationally from hospital environments.
- Understanding the distinct risk patterns in community settings can inform targeted interventions to reduce workplace violence and protect healthcare workers.

**Abstract:**

**Background**: Despite the general interest in WPV against healthcare workers, there is evidence that this topic has comparatively fewer studies conducted in the context of community settings than in hospital settings. Given the current general transition of care from hospital to community, this study aims to analyze whether community settings present different characteristics in comparison with hospital settings on this topic in Italy. **Methods**: A retrospective observational study was conducted from 2020 to 2024 on aggressions reported by HCWs in hospitals and community settings belonging to a Local Health Authority of Turin in Piedmont. For physical and non-physical aggressions, a monthly time trend series was constructed. A Mantel–Haenszel fixed-effect meta-analysis was performed to obtain the odds ratio (OR) in two settings. Variables relative to aggressions included the gender of victims, their professional category (medical doctors, nurses, other HCWs), the type and gender of perpetrators (relative, patient, or unknown person), age groups of perpetrators (under 30, 30–49, ≥50), the nature of aggression (physical, non-physical), recidivism, involvement of law enforcement, and time of occurrence (morning, afternoon, or evening/night). Events within hospitals were further classified into emergency department, psychiatric ward, and other wards, while events within community settings were classified as drug addiction service units (serDs), long-term care (including specialist outpatient services, home services, and nursing homes) (LTC), mental health centres, and penitentiary assistance. **Results**: The results highlighted that fewer WPV incidents were reported in community settings than in hospital settings, even though reported incidents showed a more pronounced increase over time. Differences were observed in a few characteristics of WPV (age classes of aggressors, recidivism, time of aggression, profession of the assaulted worker, and specific location). Only the gender of the assaulted (female workers) (OR = 3.11, 95% CI: 1.27–7.61; *p* = 0.013; OR = 0.32, 95% CI: 0.13–0.79; *p* = 0.013 for non-physical and physical violence, respectively, compared to male workers) was identified as a specific risk factor for community settings. **Conclusions**: Modern health systems are experiencing a transition from hospital-centred to community-centred care settings. This study suggested that WPV is a significant concern, even outside the hospital. Community-based services often involve direct interaction with frail and chronically ill patients and their caregivers, as well as care delivery in diverse and sometimes less controlled environments, which may influence exposure to aggressive behaviours. The identification of setting-specific risk patterns in both hospital and community contexts provides valuable insights into workplace violence and may support the planning and implementation of targeted interventions aimed at mitigating the frequency and burden of WPV.

## 1. Introduction

In recent years, workplace violence (WPV) against healthcare workers (HCWs) has become a critical concern worldwide [1,2].

WPV is defined by the WHO as “the intentional use of physical force or power, threatened or actual, against oneself, another person, group, or community, resulting in, or having a high likelihood of resulting in, injury, death and/or psychological or developmental harm or deprivation” [3]. WPV encompasses any physical, verbal, or psychological abuse, including threats, harassment, physical assault, bullying, and other forms of aggression [4]. This topic must be observed within the new organizational arrangement required by budgetary crisis and, especially, by the epidemiological transition of the population.

European health and social care systems have long been called upon to address the increasing prevalence of chronic diseases among elderly people. The Italian case is illustrative. According to ISTAT, as of 1 January 2024, individuals under the age of 15 represented 12.2% of the Italian resident population, whereas those aged over 65 accounted for 24.35% and those over 85 for more than 4.5%. Since 2000, the old-age index, that is, the ratio between those over 65 and those under 15, has risen from 1.2 to 1.95. This value brings Italy to the top of the rankings of an increasingly aging European Union, where demographic aging is accompanied by an ever-increasing incidence of chronic diseases. In Italy, at the beginning of the century, approximately 30% of the resident population was affected by at least one chronic disease. In 2023, the same percentage rose to 40.5%, and the incidence increased further with increasing age, reaching 51.6% in the 55–59 age group and 85% in the over-74 age group. The situation of multi-chronicity is also frequent: people living with more than one chronic disease are already more than a fifth of the total population [5].

Chronic pathologies require a care approach different from the management of acuity and, bolstered by the evidence-based practice, a patient-centred care model (PCCM). This raises the need to shift the delivery of care from traditional care settings such as hospitals to community care settings [6,7]; this involves numerous professional figures (e.g., doctors, nurses, rehabilitation technicians, social workers) and includes all services that intervene before and, if necessary, after hospital treatment.

In Italy, so-called “community healthcare” encompasses activities and services widespread throughout the territory, from primary care to specialist outpatient and consultative services (drug addiction service units (serDs), mental health services, etc.), from home services to nursing homes. Recent national policies have further accelerated the shift from hospital-centred to community-based care. In Italy, Ministerial Decree 70, the National Recovery and Resilience Plan (PNRR), and Ministerial Decree 77 [8] have redefined the organizational framework of health service delivery with the aim of strengthening non-hospital settings and fostering closer integration between hospital and community care.

As a result of this shift, there is a higher likelihood that healthcare workers will do their work in nursing homes, in patients’ private homes, or in outpatients settings, which poses a risk to their safety and well-being considering the dynamics and potentially dangerous factors that exist in this unpredictable setting of care. Community settings involve working alone and home visits, leading to potential reduced security, chronic/psychiatric case-mix, longer waits compared to hospital setting, overcrowding, or high acuity [9,10].

Some characteristics define the previous literature on this topic: a focus on violence perpetrated towards specific groups of HCWs (i.e., physicians, nurses, dental hygienists, or emergency medical service personnel) [7,11,12,13]. Most of the studies conducted have only addressed workplace violence in the hospital setting [14,15,16], often focusing on single wards or divisions such as emergency departments, psychiatric hospitals, and geriatric wards, where exposure to workplace violence is of particular concern [2,17,18,19,20,21]. Only more recently have studies examined workplace violence against HCWs in non-hospital settings [11,12,22], although these have generally been limited to specific settings, such as primary care [23,24,25] or home care [26], and have often been conducted in countries not conventionally identified as part of the Western world [23,24,25,27].

Despite growing interest in WPV against healthcare workers, research has largely focused on hospital settings. Evidence regarding community-based healthcare—such as home care, nursing homes, outpatient clinics, and specialized services like mental health or addiction units—is scarce [28]. Furthermore, to our knowledge, studies that directly compare hospital and community settings are absent in the Italian healthcare system.

The Italian context is an interesting case to focus on, as developments in recent years indicate growing awareness of the problem. This awareness has led to the establishment in 2022 of the National Observatory on the Safety of Healthcare Workers (GU Serie Generale n.41 of 18-02-2022) [29], the introduction of the National Day of Education and Prevention against Violence toward Healthcare Workers (12 March), and in 2019, to the ratification of the International Labour Organization Convention on Violence and Harassment (No. 190, 2019). These initiatives were aimed at monitoring incidents of violence against healthcare professionals and identifying strategies to prevent and address violence and harassment in the workplace. Understanding differences between hospital and community settings is particularly relevant because these contexts differ in patient characteristics, care delivery, and organizational arrangements, which may influence exposure to WPV. By analyzing both settings in parallel, this study aims to identify setting-specific patterns and risk factors, thereby addressing a clear knowledge gap in the Italian healthcare context.

To contribute to overcoming knowledge gaps, this study aims to analyze whether WPV in community settings presents different characteristics compared to hospital settings. Specifically, the study addresses the following research questions: (1) What are the characteristics of reported WPV incidents in community and hospital settings? (2) How did the frequency of reported WPV incidents change over time in the two settings during the 2020–2024 period? (3) Which factors are associated with different types of WPV in community and hospital settings?

## 2. Materials and Methods

A retrospective observational study from 2020 to 2024 of events classified as workplace aggressions reported from HCWs in healthcare settings was conducted from hospitals and community settings belonging to the Local Health Authority (LHA) of Turin in Piedmont. The LHA serves a population of approximately 860,000 inhabitants. It is organized into 4 hospitals (1 hub and 3 spokes) and in 4 districts, each serving approximately 200,000 residents and delivering community-based healthcare services across a large metropolitan area. The LHA included 5974 employees, of whom 993 were physicians and 2518 nurses; 1464 were men, and 4510 were women; 456 were aged <30 years, 2342 were aged 30–49 years, and 3176 were aged ≥50 years [30].

The study was based exclusively on fully anonymized incident-report data and was conducted in accordance with the applicable institutional and legal framework governing anonymized secondary data analysis. The data collection was made and overseen by the medical directorate of LHA. All data were analyzed in aggregated form and anonymized prior to sharing. As the dataset was completely anonymous and contained no identifiable personal information, formal ethical approval was not necessary. This was a quality improvement study for which the dataset was completely anonymized and contained no identifiable personal information. No written consent was therefore possible, nor was IRB approval required (D.lgs. 30 giugno 2003, n. 196). Because the dataset is based on voluntarily submitted incident reports, the observed counts may reflect reporting behaviour, as well as the underlying occurrence of events, including potential differences in reporting practices across professional groups and care settings.

### 2.1. Descriptive Analysis Framework

The prevalence and characteristics of violent episodes against HCWs were examined through descriptive analyses of categorical variables, reported as frequencies and percentages, and were categorized by place of events (hospital or community settings). Variables considered included the gender of victims, their professional category (medical doctors, nurse, other HCWs), the type and gender of perpetrators (relative, patient, or unknown person), age groups of perpetrators (under 30, 30–49, ≥50), the nature of aggression (physical, non-physical), and recidivism. Self-harm and deprivation episodes were not reported. Additional indicators included the involvement of law enforcement and the time of occurrence (morning, afternoon, or evening/night). Events within hospitals were further classified into emergency department, psychiatric ward, or other wards. Events within community settings were classified as drug addiction services unit (serD), long-term care (including specialist outpatient, home services, and nursing homes) (LTC), mental health centre, or penitentiary assistance. This classification reflects the framework defined by Ministerial Decree 77 (DM 77) [31].

### 2.2. Time Trend Analysis

For each aggression type (physical, non-physical), monthly time series were constructed aggregating data by calendar month and year. Temporal patterns were examined using ordinary least squares (OLS) regression models in which time was represented by a continuous monthly index.

Two nested model specifications were applied. The first assessed whether the outcome exhibited a consistent linear change across months by modelling the outcome as a function of the monthly time index. This allowed estimation of the average month-to-month trend and provided a formal test of whether that trend differed from zero. The second specification extended the model by adding a quadratic time term to capture potential nonlinear features such as accelerating, decelerating, or otherwise curved trajectories over the study period.

Model estimation relied on OLS, which derives coefficient estimates by minimizing the sum of squared differences between observed monthly values and model-predicted values. Inference for each coefficient was based on two-sided *t*-tests, using standard errors consistent with the assumptions of the regression framework. These tests evaluated whether each time-related parameter differed significantly from zero.

In the linear specification, the key hypothesis test concerned the monthly trend term, determining whether a statistically meaningful directional change occurred over time. In the quadratic specification, separate *t*-tests were performed for both the linear and quadratic components. The linear component reflected the overall direction of change, while the quadratic component assessed whether the rate of change varied across the time series. A statistically significant quadratic term was interpreted as evidence that the outcome followed a nonlinear rather than a strictly linear trajectory. For each model, the resulting coefficient estimates, standard errors, t-statistics, and *p*-values were used to assess the magnitude and statistical reliability of the temporal pattern.

Graphical summaries were generated by plotting monthly values across the study period and superimposing both linear and quadratic fitted curves. These visualizations complemented the statistical tests by illustrating the shape and consistency of the estimated time trends.

### 2.3. “Typical Month” Profile (Month-Averaged Pattern)

To characterise the average behaviour, we constructed a descriptive month-averaged profile. For each calendar month (January, February, …, December), we computed the mean of the monthly counts across all available years. Concretely, for a given month:

- all observations corresponding to month across different years were identified;
- their arithmetic mean was calculated.

This procedure yields a set of twelve month-specific averages that represent an “average year” profile of the outcome, smoothing out year-to-year noise and emphasizing systematic variation. These month-averaged values were used descriptively to identify months with consistently higher or lower burden.

### 2.4. Risk Analysis and Stratified Meta-Analysis

To analyse binary outcomes (e.g., presence/absence of a specific type of aggression), we constructed 2 × 2 contingency tables comparing exposure groups and reference categories, with and without stratification by additional covariates.

For a given outcome type (e.g., a specific aggression category), the binary variable was defined as:

- event = 1 if the outcome variable equalled the type of interest;
- event = 0 otherwise.

The main exposure variable (e.g., profession, labelled generically as group_col) was treated as a factor with one prespecified reference level (e.g., sex: male). For each non-reference level (e.g., female), we constructed 2 × 2 tables of:

- rows: exposure group (level versus reference);
- columns: event versus non-event.

When stratification was required (e.g., by sex, profession location, or other covariates), strata were defined by the combination of the chosen stratifying variables. For each stratum, a separate 2 × 2 table was computed for the contrast “level versus reference”.

For each contrast, we then performed a Mantel–Haenszel fixed-effect meta-analysis across strata to obtain a pooled odds ratio (OR) and its 95% confidence interval. Mantel–Haenszel pooling was preferred to account for stratification while assuming a common underlying effect across strata. In the presence of zero cell counts, a Haldane–Anscombe continuity correction (adding 0.5 to all cells in the affected table) was applied to ensure stable estimation on the log scale. Forest plots were used to display stratum-specific ORs and the pooled Mantel–Haenszel estimate.

We also computed 95% confidence intervals and two-sided *p*-values derived from Wald-type tests on log (OR). When any cell contained zero counts, a continuity correction (Haldane–Anscombe 0.5) was again applied. Confidence intervals and *p*-values were calculated on the log scale and then back-transformed for reporting. Results are presented in summary tables listing number of events, number of non-events, total, and OR (with 95% CI and *p*-value) for each level; these were reported separately for each main subgroup.

### 2.5. Software

All analyses were conducted using R (R Foundation for Statistical Computing, Vienna, Austria; version 4.5.3). Ordinary least squares regression was performed using base stats functions, with coefficient testing supported by lmtest (version 0.9-40) and heteroskedasticity-consistent standard errors from sandwich (version 3.1-1), where appropriate. Mantel–Haenszel risk-ratio meta-analyses and forest plots were carried out using the meta package (version 8.2-1), with metafor (version 4.8-0) used when needed; tabulation of event counts relied on stats and dplyr (version 1.2.0). Manual implementations of Mantel–Haenszel formulas used base stats functions. Graphical displays, including fitted trend curves and residual diagnostics, were generated using graphics and ggplot2 (version 4.0.2). Summary tables were produced with R Markdown-compatible tools, including rmarkdown (version 2.31). A two-sided significance level of α = 0.05 was adopted throughout.

## 3. Results

### 3.1. Descriptive Findings

A total of 516 notifications were collected between 2020 and 2024, with around 30% of incidents occurring in community settings (n = 151). Over the study period, the proportion of employees with at least one reported WPV episode was 8.63% overall and 10.17% among physicians, 11.79% among nurses, 10.66% among men, and 7.43% among women. In both settings, most of the assaults were non-physical and did not require law enforcement intervention. The assaults were mostly in LTC in the community and in psychiatric wards in hospitals. Most of the aggressions occurred in the morning (55%) in community settings, while in hospitals, mostly in the afternoon and evening/night (67.9%). Perpetrators were mostly male and predominantly patients in both settings (65.6% vs. 66.6%; 68.9% vs. 75.9% in community settings and hospitals, respectively); the perpetrators’ profile differed in relation to age—with aggressors more frequently aged ≥50 years in community settings and younger in hospitals (<30 years)—and in recidivism, with a greater proportion of non-repeat aggressors in community settings and of habitual aggressors in hospitals.

A relatively high proportion of reports included missing information for some aggressor characteristics, particularly aggressor age and aggressor type. These missing values likely reflect the fact that healthcare workers may not always have complete information about the aggressor at the time of reporting.

Victims were mostly female in both settings (68.9% vs. 63.3% in community settings and hospitals, respectively), and in community settings, physicians were the most frequently affected professional group (39.1%), whereas in hospital settings, nurses were the most frequently affected professional group (65.8%).

Detailed characteristics of the workers assaulted, the aggressive acts, and the aggressors are summarized in Table 1.

### 3.2. Time Series Analysis and Risk Analysis

**Figure 1:**
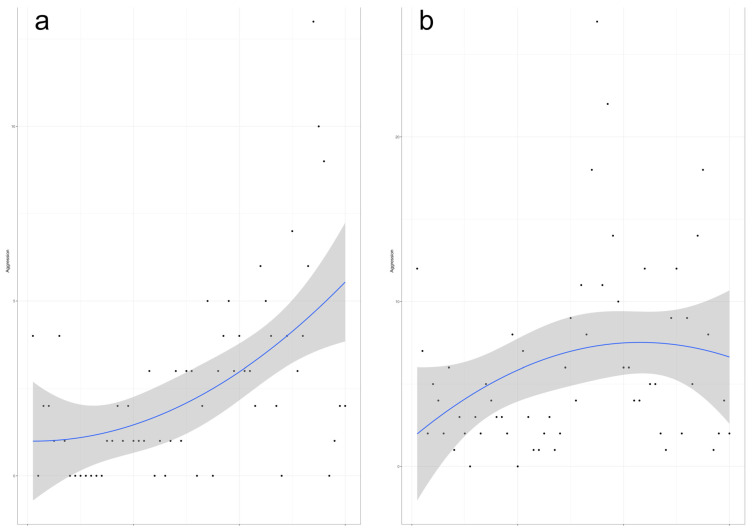
Quadratic trends in aggressions in community and hospital settings, 2020–2024. Note: (**a**)—community settings, (**b**)—hospital settings. Black dots indicate observed monthly counts; the blue line, the fitted quadratic trend; and the grey shaded area, the 95% confidence interval.

In community settings, the linear time trend was positive and statistically significant, indicating a month-to-month increase in aggressions over the study period (coefficient for the linear term = +0.92, 95% CI: 0.52–1.33), whereas the quadratic trend was not significant (coefficient for the quadratic term = 0.02, 95% CI: −0.01–0.04) (Figure 1).

In hospital settings, the linear coefficient was also positive, indicating an average month-to-month increase over the 5-year study period (coefficient for the linear term = 0.95; 95% CI: −0.02–1.92); however, this increase was not statistically significant. The quadratic term was negative but also not statistically significant (coefficient for the quadratic term = −0.04; 95% CI: −0.10–0.02).

**Figure 2:**
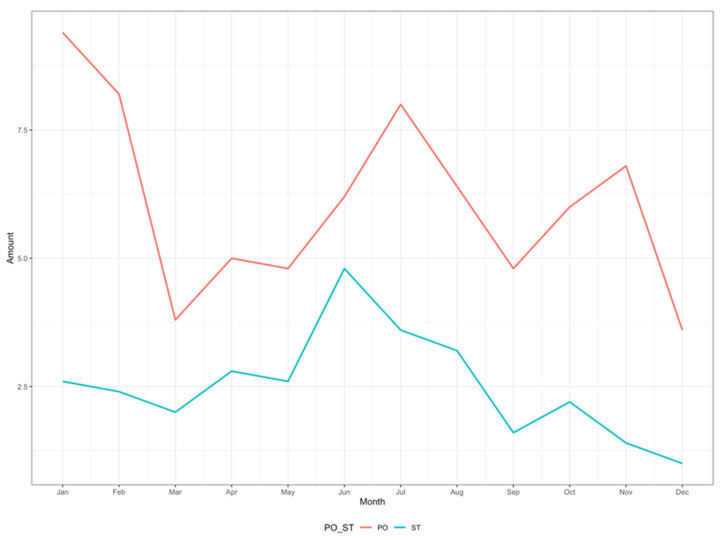
Trend in monthly aggressions in hospital and community settings.

Figure 2 shows a monthly distribution of aggressions, with a higher incidence in the months of June and July, in both settings, together with an additional peak in the month of January for the hospital setting only.

In community settings, the risk analysis indicated higher odds of non-physical aggression among females (OR = 3.11, 95% CI: 1.27–7.61; *p* = 0.013) and during the afternoon (OR = 14, 95% CI: 2.51–78; *p* = 0.003; note that low data count may bias the result). Conversely, moreover, females had lower odds of physical aggression (OR = 0.321, 95% CI: 0.13–0.79; *p* = 0.013), as did incidents occurring in the morning (OR = 0.071, 95% CI: 0.01–0.40; *p* = 0.003) (Table 2).

In hospital settings, workers were significantly more exposed to physical aggression in psychiatric wards and emergency departments (OR = 6.75, 95% CI: 3.55–12.80; *p* < 0.01; OR = 2.92, 95% CI: 1.46–5.82; *p* = 0.02, respectively), while the odds of non-physical violence were lower in these wards (OR = 0.148, 95% CI: 0.08–0.28; *p* < 0.01; OR = 0.343, 95% CI: 0.17–0.68; *p* = 0.02, respectively). Patients had substantially higher odds of engaging in physical aggression (OR 7.62, 95% CI: 3.31–17.60; *p* < 0.01) and lower odds of non-physical violence (OR 0.131, 95% CI: 0.06–0.30; *p* < 0.01) (Table 2).

## 4. Discussion

This study investigated whether WPV in community settings differed from that observed in hospital settings, addressing three main questions: what were the characteristics of reported WPV incidents in community and hospital settings? How did the frequency of reported WPV incidents change over time in the two settings during the 2020–2024 period? Which factors are associated with different types of WPV in community and hospital settings?

The results highlighted that fewer WPV incidents were reported in community settings than in hospital settings, although the increasing trend appeared more pronounced in community settings. There were differences in a few characteristics of WPV (age classes of aggressors, recidivism, time of aggression, profession of the assaulted worker, and specific location); only the gender of the assaulted worker (female) and time of aggressions were identified as specific risk factors for community settings.

In the community settings covered by the LHA, from 2020 to 2024, among 516 reported incidents, 29.3% occurred in community settings. This highlights that WPV is a systemic problem that is not limited to hospitals but also affects community settings [7,32]. In both care settings, the majority of reported incidents were non-physical, with a higher proportion in community contexts than in hospitals. The higher frequency of non-physical violence compared to physical violence observed in our study is a constant feature in analyses of WPV: this distribution is reported in several reviews and meta-analyses and appears to be consistent worldwide [1,33,34]. Physical violence, although less frequent than non-physical violence, is still a significant problem considering the number of cases involved. As a case in point, in the systematic review by Liu et al., the 12-month prevalence of physical violence against HCWs in Europe was 20.1%. (95% CI: 15.3–25.2%) [1]. In Berger et al.’s systematic review and meta-analysis, physical violence in intensive care units was reported with a median proportion of 43% (IQR 27–70%) in the eight European studies included in their analysis [34]. In our study, instead, the percentage of physical violence compared to the total number of incidents was 19.9% in the territorial context and 47.4% in the hospital context. However, direct comparisons with survey-based prevalence estimates should be interpreted cautiously, as our data reflect reported incidents rather than survey-reported person-level exposure.

Regarding the gender of the victims, women accounted for the highest percentage of HCWs assaulted in both settings, although gender differences in the hospital context did not reach statistical significance. Professional groups most frequently affected by WPV vary by setting and profession. Unexpectedly, physicians were the most exposed in community settings, predominantly in long-term care facilities. On the other hand, nurses were the most exposed in hospitals, most frequently in psychiatric wards and emergency departments. Perpetrators were predominantly male and were patients in both settings. The results relating to the gender and category of victims and aggressors in hospitals confirm what has already been reported in Italian studies, well summarized in a scoping review [35]. On the other hand, the analysis of these patterns in the community context fills a gap in the literature that has not yet been explored in Italy.

Our results showed that aggressor age and recidivism vary depending on the context: perpetrators were older and predominantly non-recurrent in community settings, while they were younger and recurrent aggressors in hospitals. These findings are consistent with the hypothesis that perpetrator characteristics may, at least in part, mirror the case-mix of patients served in community settings; however, this mechanism cannot be empirically evaluated in our dataset. The available data seems to suggest that the average age of people attending community settings may be higher than that of people attending hospitals, although in hospital settings, it is particularly difficult to estimate. With regard to community care in Italy, for example, as of 1 January 2024, more than 75% of residents in nursing home facilities were over 65 years old, 19% were between 18 and 64 years old, and the remaining 6% were under 18 years old [36]. In the field of homecare assistance, according to the Ministry of Health website, patients aged 65 and over represent more than 84% of the total number of patients assisted in integrated homecare [37]. Moreover, according to the epidemiological observatory on addiction in Piedmont, the average age of serD users in 2023 was 46.3 years [38]. Finally, according to the Italian Ministry of Health’s mental health report, the highest concentration of patients in mental health centres in 2023 was in the 45–54 and 55–64 age groups [39]. However, the abovementioned hypothesis remains speculative and should be formally examined in future studies.

Given the descriptive and exploratory nature of this analysis, and limitations of the modelling approach, the results of time series analysis should be interpreted as indicative patterns rather than formal inferential estimates. In this premise, time series analysis revealed some interesting characteristics. Unlike the trend in hospital settings, in community settings, WPV showed a statistically significant month-over-month increase, while the evidence relating to curvature/acceleration was limited and not significant. Our results seemed to confirm that WPV also affects community settings [7,32] and add a time perspective showing an increase in reported incidents in recent years. However, given that our data are based on victim-submitted incident reports, this trend may reflect under-reporting, changes in reporting practices, a true rise in incidence, or a combination of all these factors [40]. It remains to be understood whether this increase is related to the characteristics of the patients assisted, the peculiarities of the territory, improvements in detection capacity, or reduced under-reporting over time [7,41].

The month-averaged distribution of events observed may reflect a combination of organizational factors within the service and month-averaged variations in demand. The June–July increase across both hospital and community settings could be consistent with summer holiday-related staff shortages and facility closures or service reconfigurations. According to a survey conducted by the Federation of Associations of Hospital Internist Managers (FADOI) and analyzing 206 hospital wards of internal medicine throughout Italy, more than 91% of physicians take their 15 days of holiday during the summer period: this leads to reductions in staffing levels in wards and outpatient activities, which decrease in 52.7% of cases [42]. Data on the territorial context are lacking, but a reorganization or suspension of supply during the summer period is usual. In general, the resulting reduced capacity to absorb demand and longer waiting or management times could exacerbate patients’ dissatisfaction, contributing to WPV against HCWs [7,33,43]. The additional spike in WPV recorded in January, which was limited to hospitals, could be related to the seasonal increase in the circulation of respiratory viruses, which leads to increased work pressure on hospital staff and services and greater difficulties in meeting patients’ needs. However, in the absence of staffing, activity-volume, and waiting-time data, mechanistic explanations for the month-averaged pattern remain hypothesis-generating and cannot be tested in this study.

Risk analysis identified setting-specific determinants. In the territorial context, female HCWs were substantially more likely to experience non-physical aggression and less likely to experience physical aggression than their male colleagues. These results seem to confirm a pattern observed by some studies in the hospital setting and extend it to the community settings [44,45,46]. According to our findings, the risk of violence would also vary markedly by time of day: the likelihood of physical aggression was significantly lower in the morning than during evening and night, whereas the likelihood of non-physical violence was markedly higher in the afternoon compared with evening and night. These results should be taken with caution, as they may be biased by a low data count; however, it can be assumed that the above-mentioned pattern is related to the type and condition of the patients, as well as the specific setting. The literature confirms that morning is less interspersed with aggressions. In a review of Gillespie, several studies conducted worldwide have shown that WPV is more frequent at certain times of the day, particularly during the afternoon, evening, and night [47]. However, without patient-level severity and key operational metrics, time-of-day mechanisms remain speculative and cannot be tested in this study. Nonetheless, the observed pattern may be compatible with variation in the patient case-mix and clinical status across shifts, as well as setting-specific service organization.

Notably, this study did not report any risk factors inherent to the type of aggressor or the location of the aggression in community settings, unlike the hospital settings.

It is true that our results highlighted that there were not specific community settings at greater/lesser risk of aggression, but it is equally true that they do not exclude the fact that all territorial settings were affected by the phenomenon of aggression. The existing literature agrees that assaults are also perpetrated in psychiatric outpatient units and not only in psychiatric wards—with verbal violence being more frequent than non-verbal [22,48,49]—as well as in outpatient units treating substance use disorders, highlighting a close relationship between alcohol or drug abuse and violent behaviour [50,51]. Similarly, WPV has plagued homecare and nursing home settings [52,53]. Studies of resident-to-staff aggression in nursing homes found the phenomenon to be highly prevalent, and the most common behaviours were verbal aggression [54,55]; a review reported that verbal abuse, being the most common form of patient aggression, is estimated to be perpetrated on anywhere between 33% and 87% of home care staff [6].

The absence of risk factors inherent to the specific community setting may certainly reflect limited statistical power, heterogeneous reporting practices, or residual under-reporting that may conceal an uneven distribution of violence. However, given the absence of evidence for setting-specific gradients in our data, the risk of WPV in territorial services should be considered across all community locations and user groups. Similarly, the lack of an association with perpetrator type in community services should not be interpreted as evidence that perpetrator profiles are irrelevant; rather, it indicates that our data did not detect differences across categories. We expected perpetrator type to vary across services, in relation to the type of activity and patient, but this could not be confirmed within the present models. In home care, caregiver-perpetrated aggression related to caregiver burden remains a plausible interpretation that cannot be empirically evaluated here.

In contrast, in the hospital setting, the risk of physical assault was markedly higher in emergency departments (OR = 2.92, 95% CI: 1.46–5.82; *p* < 0.01) and psychiatric wards (OR 6.75, 95% CI: 3.55–12.80; *p* < 0.01) than in all other wards. This pattern mirrors observational evidence from Italian and European studies and points to a risk gradient, with violence clustering in units characterized by high clinical acuity, pronounced behavioural instability, crowding, and intense emotional pressure [35,56,57,58].

The profile of perpetrators also differed by type of aggression: the likelihood that non-physical assaults were perpetrated by patients is substantially lower than that for caregivers; regarding physical violence, the odds that the aggressor was a patient were almost eight times higher than for caregivers. This pattern is partly consistent with the evidence synthesized by Civilotti et al.’s scoping review, which reports that among studies distinguishing patients from visitors, patients are consistently identified as primary aggressors, particularly for physical assaults [35].

This study presents the inherent limitations of observational research conducted in a single metropolitan area, which limits its generalizability. Moreover, as the analysis was based on incident reports voluntarily submitted by victims, temporal variations may partly reflect changes in reporting behaviour or awareness rather than true changes in incidence. However, a key strength is the systematic and parallel analysis of hospital and community settings; a perspective that, to our knowledge, is not available in Italian studies. According to the literature, specific community settings are investigated [7], or specific departments including multiple services, (e.g., inpatient, outpatient rehabilitation [49]), but not multiple settings simultaneously or in a directly comparative framework. General practitioners were not included, as no structured data were available as of 2020; however, all other components of community care were included. Furthermore, the study was conducted in a single metropolitan area, so our findings may not fully reflect WPV patterns across all components of community care in Italy. We were unable to incorporate key organizational and contextual determinants (staffing levels, overcrowding, workload, burnout) or direct indicators of downstream impact (sickness absence, turnover intention, perceived quality of care). In addition, information on the distribution of personnel by professional category within hospital and community settings was not available in the administrative dataset. As a result, it was not possible to calculate profession-specific incidence rates using appropriate denominators. However, the integration of temporal dimensions (month, time of day) and department type allowed us to describe spatial–temporal risk patterns in great detail and formulate hypotheses for targeted prevention.

Our findings indicate several priorities for future research. Multicentre studies are needed to confirm the reproducibility of this study’s findings, including also organizational variables and consequences of aggressions. At the same time, preventive measures should be evaluated to assess their effectiveness.

## 5. Conclusions

In conclusion, modern health systems are experiencing a transition from hospital-centred to community-centred care settings. This study supported the assumption that assault is a significant concern even outside the hospital. Community-based services often involve direct interaction with frail and chronically ill patients and their caregivers, as well as care delivery in diverse and sometimes less controlled environments, which may influence exposure to aggressive behaviours [59]. Although incidents were reported less frequently in community settings than in hospitals, reported cases seem to show an increase over time. Given the nature of the data source, this trend may reflect changes in reporting behaviour, a true rise in incidence, or a combination of both. The identification of setting-specific risk patterns in both hospital and community contexts provides valuable insights into workplace violence and may inform future research, surveillance, and prevention efforts aimed at reducing the frequency and burden of WPV in Italy.

## Figures and Tables

**Table 1:** Characteristics of the aggressive acts, the aggressors, and the workers assaulted. Unknown categories were reported descriptively but excluded from the corresponding analytical models.

		Community Setting		Hospital Setting		Total	
		N.	%	N.	%	N.	%
**Characteristics of the aggressive acts and the aggressors**							
Number of aggressions		151	29.3	365	70.7	516	
Types of aggressions	Physical	30	19.9	173	47.4	203	39.3
	Non-physical	121	80.1	192	52.6	313	60.7
Location of aggressive acts							
Hospital setting-location	Emergency department			101	27.7	101	19.6
	Other wards			74	20.3	74	14.3
	Psychiatric ward			182	49.9	182	35.3
	Unknown			8	2.2	8	1.6
Community setting-location	Drug addiction service unit (serDs)	30	19.9			30	5.8
	Long-term care	59	39.1			59	11.4
	Mental health centre	26	17.2			26	5
	Penitentiary assistance	18	11.9			18	3.5
	Unknown	18	11.9			18	3.5
Time of aggressive acts	Morning	83	55	114	31.2	197	38.2
	Afternoon	60	39.7	125	34.2	185	35.9
	Evening and night	8	5.3	123	33.7	131	25.4
	Unknown	0	0	3	0.8	3	0.6
Types of aggressors	Patients	104	68.9	277	75.9	381	73.8
	Relatives/caregivers	19	12.6	49	13.4	68	13.2
	Unknown	28	18.5	39	10.7	67	13
Sex	Female	32	21.2	87	23.8	119	23.1
	Male	99	65.6	243	66.6	342	66.3
	Unknown	20	13.2	35	9.6	55	10.7
Age classes	<30 years	20	13.2	96	26.3	116	22.5
	30–49 years	37	24.5	86	23.5	123	23.8
	≥50 years	46	30.5	86	23.5	132	25.6
	Unknown	48	31.8	97	26.6	145	28.1
Intervention of security forces	No	82	54.3	258	70.7	340	65.9
	Yes	48	31.8	73	20	121	23.4
	Unknown	21	13.9	34	9.3	55	10.7
Recidivism	No recidivism	68	45	112	30.7	180	34.9
	Recidivism	47	31.1	158	43.3	205	39.7
	Unknown	36	23.8	95	26	131	25.4
**Characteristics of the workers assaulted**							
Sex	Female	104	68.9	231	63.3	335	64.9
	Male	39	25.8	117	32.1	156	30.2
	Unknown	8	5.3	17	4.7	25	4.8
Profession	Nurse	57	37.7	240	65.8	297	57.6
	Medical doctor	59	39.1	42	11.5	101	19.6
	Other healthcare workers	32	21.2	74	20.3	106	20.5
	Unknown	3	2	9	2.5	12	2.3

**Table 2:** Risk factors of healthcare workers assaulted in community and hospital settings. Strata reference for each group are reported as in Table 1.

Community Setting							
		Non-Physical WPV	95% CI	*p*	Physical WPV	95% CI	*p*
**Characteristics of the workers assaulted**							
Sex	Female	3.11	1.27–7.61	0.013	0.32	0.13–0.79	0.013
	Male	1			1		
Profession	Nurse	1.08	0.35–3.32	0.087	0.92	0.30–2.83	0.887
	Medical doctor	0.82	0.28–2.40	0.713	1.23	0.42–3.61	0.713
	Other healthcare workers	1			1		
**Characteristics of the aggressive acts and the aggressors**							
Location of aggressive acts							
Hospital setting (location)	Emergency department						
	Other wards						
	Psychiatric ward						
Territorial setting (location)	Drug addiction service unit (serDs)	2.50	0.63–9.86	0.191	0.40	0.10–1.58	0.191
	Long-term care	3.19	0.93–10.90	0.065	0.31	0.09–1.07	0.065
	Mental health centre	1.67	0.44–6.36	0.455	0.60	0.16–2.29	0.046
	Penitentiary assistance	1			1		
Time of aggressive acts	Morning	2.77	0.64–12.00	0.174	0.07	0.01–0.40	<0.01
	Afternoon	14	2.51–78.00	<0.01	0.36	0.08–1.57	0.174
	Evening and night	1			1		
Types of aggressors	Patients	0.20	0.03–1.54	0.122	5.11	0.65–40.4	0.122
	Relatives/caregivers	1			1		
Sex	Female	1			1		
	Male	1.18	0.44–3.13	1.741	0.85	0.32–2.25	0.741
Age classes	<30 years	2.48	0.47–13.00	0.282	0.40	0.08–2.11	0.282
	30–49 years	1			1		
	≥ 50 years	0.88	0.31–2.47	0.805	1.14	0.41–3.21	0.805
**Hospital Setting**							
		**Non-Physical WPV**	**95% CI**	***p***	**Physical WPV**	**95% CI**	***p***
**Characteristics of the workers assaulted**							
Sex	Female	1.18	0.76–1.84	0.469	0.85	0.54–1.32	0.469
	Male	1			1		
Profession	Nurse	1.44	0.86–2.44	0.169	0.69	0.41–1.17	0.169
	Medical doctor	2.02	0.93–4.38	0.075	0.50	0.23–1.07	0.075
	Other healthcare workers	1			1		
**Characteristics of the aggressive acts and the aggressors**							
Location of aggressive acts							
Hospital setting (location)	Emergency department	0.34	0.17–0.68	0.002	2.92	1.46–5.82	<0.01
	Other wards	1			1		
	Psychiatric ward	0.15	0.08–0.28	<0.01	6.75	3.55–12.80	<0.01
Territorial setting (location)	Drug addiction service unit (serDs)						
	Long-term care						
	Mental health centre						
	Penitentiary assistance						
Time of aggressive acts	Morning	0.70	0.42–1.17	0.174	1.43	0.86–2.38	0.174
	Afternoon	1.10	0.67–1.81	0.712	0.91	0.55–1.50	0.712
	Evening and night	1			1		
Types of aggressors	Patients	0.13	0.06–0.30	<0.01	7.62	3.31–17.60	<0.01
	Relatives/caregivers	1			1		
Sex	Female	1			1		
	Male	1.39	0.85–2.28	0.187	0.72	0.44–1.17	0.187
Age classes	<30 years	0.89	0.50–1.60	0.709	1.12	0.62–2.01	0.709
	30–49 years	1			1		
	≥ 50 years	1.68	0.92–3.06	0.094	0.60	0.33–1.09	0.094

## Data Availability

Data available on request due to ethical restrictions.

## Abbreviations

The following abbreviations are used in this manuscript:

WPV	Workplace violence
HCW	Healthcare worker
serD	Drug addiction services unit
LTC	Long-term care
PCCM	Patient-centred care model
LHA	Local Health Authority
